# Hypomethylation Causes *MIR21* Overexpression in Tumors

**DOI:** 10.1016/j.omto.2020.05.011

**Published:** 2020-05-26

**Authors:** Jun Lu, Ting Tan, Ling Zhu, Huiyue Dong, Ronghua Xian

**Affiliations:** 1 Fuzhou General Clinical College, Fujian Medical University, Fuzhou, China; 2 900 Hospital of the Joint Logistics Team, Fuzhou, China; 3 Fujian Provincial Key Laboratory of Transplant Biology, Dongfang Hospital (900 Hospital of the Joint Logistics Team), Xiamen University, Fuzhou, China

**Keywords:** MIR21, hypomethylation, CpG locus, DNA demethylase, transcription factor

## Abstract

miR-21 is an oncogenic microRNA (miRNA) that is upregulated in many solid tumors. However, the effect of *MIR21* hypomethylation on miR-21 expression in tumors and the mechanism of miR-21 DNA demethylation remain unclear. In this study, we confirmed that the expression of miR-21 was significantly increased in multiple tumors. We analyzed eight types of cancer, including breast cancer (BRCA), lung adenocarcinoma (LUAD), renal and renal clear cell carcinoma (KIRC), bladder urothelial carcinoma (BLCA), hepatocellular carcinoma (LIHC), lung squamous cell cancer (LUSC), renal papillary cell carcinoma (KIRP), and pancreatic adenocarcinoma (PAAD). *MIR21* DNA methylation levels were elevated in these cancers. CpG loci located approximately 200 bp upstream of the transcription initiation site strongly affect MIR21 expression. We also confirmed *MIR21* hypomethylation by pyrosequencing of fresh clear cell renal cell carcinoma (ccRCC) samples. Demethylating agent was proved to increase hsa-miR-21-5p level in HEK293T cells, while knockdown of DNA demethylases TET3 and TDG decreased *MIR21* expression. In addition, we showed that the cg02515217 CpG locus in *MIR21* promoter was a conserved binding site of transcription factors CEBPB, MEIS3, and TEAD4, which were co-expressed with miR-21 in tumors. These observations identified that gene hypomethylation regulated the expression of *MIR21* in tumors.

## Teaser

miR-21 is an oncogenic miRNA that is upregulated in many solid tumors. In this study, we show that the *MIR21* DNA methylation levels were elevated in eight major types of cancer. Two CpG sites closely related to *MIR21* expression. One of them is the binding site of transcription factors.

## Introduction

MicroRNAs (miRNAs) are non-coding RNAs of approximately 21–25 bases in length. miRNAs exert a negative regulatory effect through complementary binding of the 3′ untranslated regions (3′ UTRs) of the target gene, either causing target gene mRNA degradation or translational inhibition. miRNA is closely related to cell proliferation, differentiation, and apoptosis, especially the occurrence and development of tumors.^1^

miR-21 is an oncogenic miRNA that is upregulated in many solid tumors (e.g., liver, lung, esophageal, and head and neck cancers) and non-solid tumors (e.g., chronic lymphocytic leukemia and B cell lymphoma).^2^ Overexpressed miR-21 is closely related to the growth, invasion, and metastasis of tumor cells.^3^ For example, miR-21 reduces phosphatase and tensin homolog (PTEN) expression and enhances hepatocellular carcinoma (LIHC) cell proliferation and invasion.^4^ Tropomyosin 1 (TPM1) was confirmed to be a target gene of miR-21 in breast cancer cells by fluorescence reporter gene analysis and western blot.^5^ The expression level of miR-21 is related to the p53 tumor suppressor gene.^6^

miRNA expression is regulated by both transcription factors and epigenetics.^7^ DNA methylation is an important way of epigenetic modification, affecting gene expression and cell development without changing the DNA sequence. DNA methylation can occur within the gene bodies, transcription initiation sites with or without the CpG island, and at regulatory elements and repeat sequences.^8^ In many tumor tissues, the specific tumor suppressor gene is hypermethylated, leading to abnormally low expression, or the oncogene is hypomethylated, leading to abnormally high expression.^9^ The Cancer Genome Atlas (TCGA) research reported that overexpression of miR-21 is associated with hypomethylation of the *MIR21* promoter in clear cell renal cell carcinoma (ccRCC)^10^ and papillary thyroid carcinoma (PTC).^11^ However, there are currently few reports on *MIR21* methylation and CpG locus that affect *MIR21* expression in other tumors.

In this study, the results of bioinformatics analysis showed a hypomethylation pattern in *MIR21* from eight major types of tumors and found two CpG sites closely related to *MIR21* expression according to TCGA data. Methylation sequencing was performed to detect *MIR21* DNA methylation in fresh renal clear cell carcinoma tissue samples collected at our center, and the correlation between methylation and gene expression was proved. Finally, DNA demethylases and transcription factors were further shown to be involved in *MIR21* expression.

## Results

### MIR21 Expression in TCGA Data

**Figure 1:**
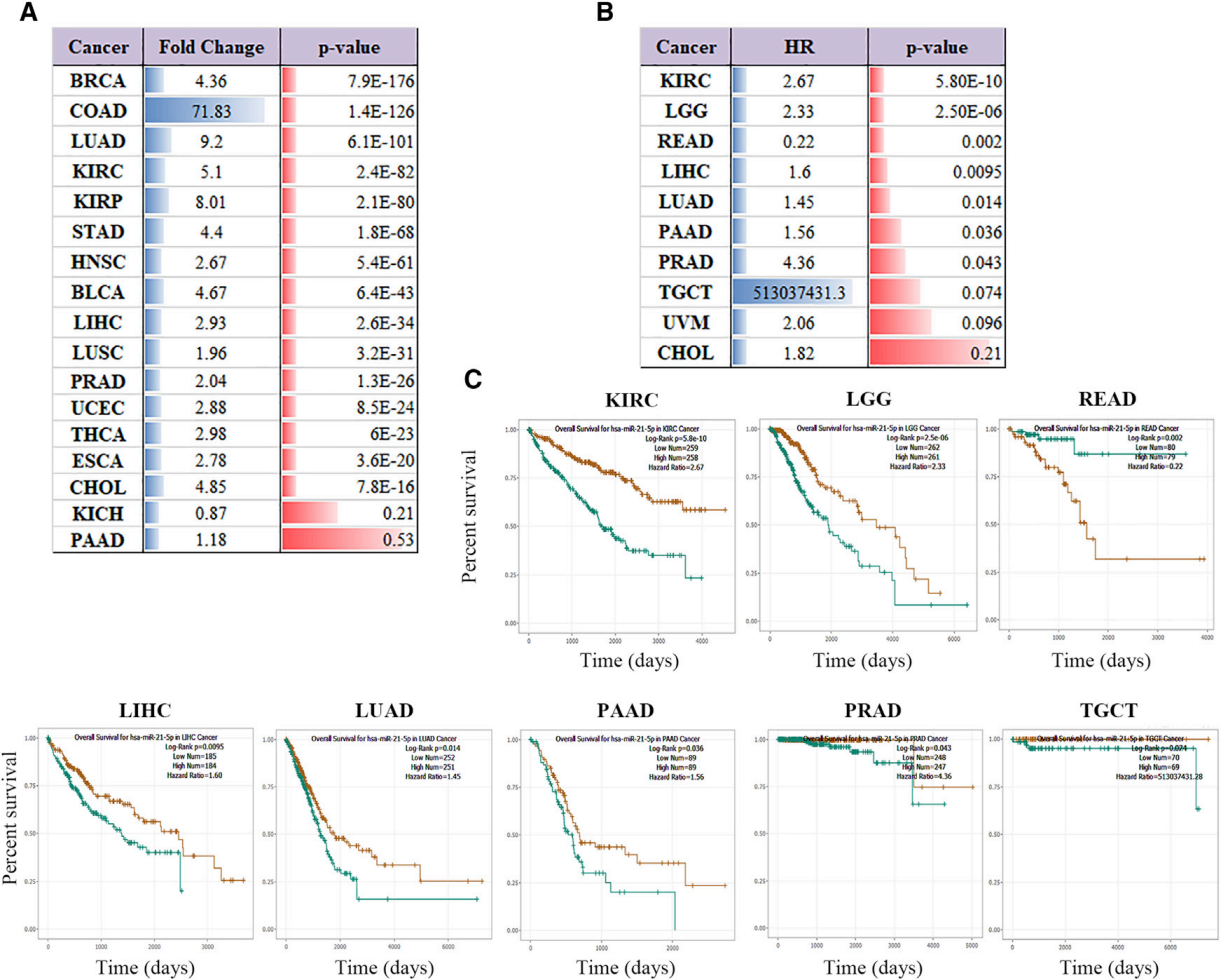
Expression Pattern and Clinical Significance of hsa-miR-21-5p in TCGA Tumors (A) Results of differential expression patterns of hsa-miR-21-5p in TCGA (tumor versus normal) samples. BRCA, breast cancer; COAD, colon adenocarcinoma; LUAD, lung adenocarcinoma; KIRC, kidney renal clear cell carcinoma; KIRP, kidney renal papillary cell carcinoma; STAD, stomach adenocarcinoma; HNSC, head and neck squamous cell carcinoma; BLCA, bladder urothelial carcinoma; LIHC, hepatocellular carcinoma; LUSC, lung squamous cell carcinoma; PRAD, prostate adenocarcinoma; UCEC, uterine corpus endometrial carcinoma; THCA, thyroid carcinoma; ESCA, esophageal carcinoma; CHOL, cholangio carcinoma; KICH, kidney chromophobe; PAAD, pancreatic adenocarcinoma. Fold change is the ratio of gene expression levels in tumor tissue to those in normal adjacent tissue. (B) Results of survival analysis of hsa-miR-21-5p in TCGA samples. LGG, brain lower grade glioma; UVM, uveal melanoma; HR, hazard ratio. (C) Kaplan-Meier analysis of overall survival for TCGA-KIRC, TCGA-LGG, TCGA-READ, TCGA-LIHC, TCGA-LUAD, TCGA-PAAD, TCGA-PRAD, and TCGA-TGCT patients relative to expression levels of hsa-miR-21-5p.

To demonstrate the expression of *MIR21* in tumors and its relationship with tumor prognosis, we downloaded the expression analysis data of hsa-miR-21-5p in 17 cancer types from the starBase website and the correlation data for hsa-miR-21-5p expression and survival time of patients with tumor. As shown in Figure 1A, hsa-miR-21-5p is highly expressed in 15 of 17 common tumors (p < 0.05). There were seven tumors in which hsa-miR-21-5p expression was associated with patient survival (Figure 1B). Among six cancer types, including kidney renal clear cell carcinoma (KIRC), brain lower grade glioma (LGG), LIHC, lung adenocarcinoma (LUAD), pancreatic adenocarcinoma (PAAD), and prostate adenocarcinoma (PRAD), low hsa-miR-21-5p was significantly associated with better survival compared with patients with high hsa-miR-21-5p expression (Figure 1C; hazard ratio [HR] > 1, p < 0.05). These expression and survival analyses indicated that miR-21 is highly expressed in most types of tumors, and higher expression of *MIR21* is a predictor of poor survival for patients with cancer.

### The Relationship between High Expression of miR-21 and DNA Hypomethylation of MIR21

**Figure 2:**
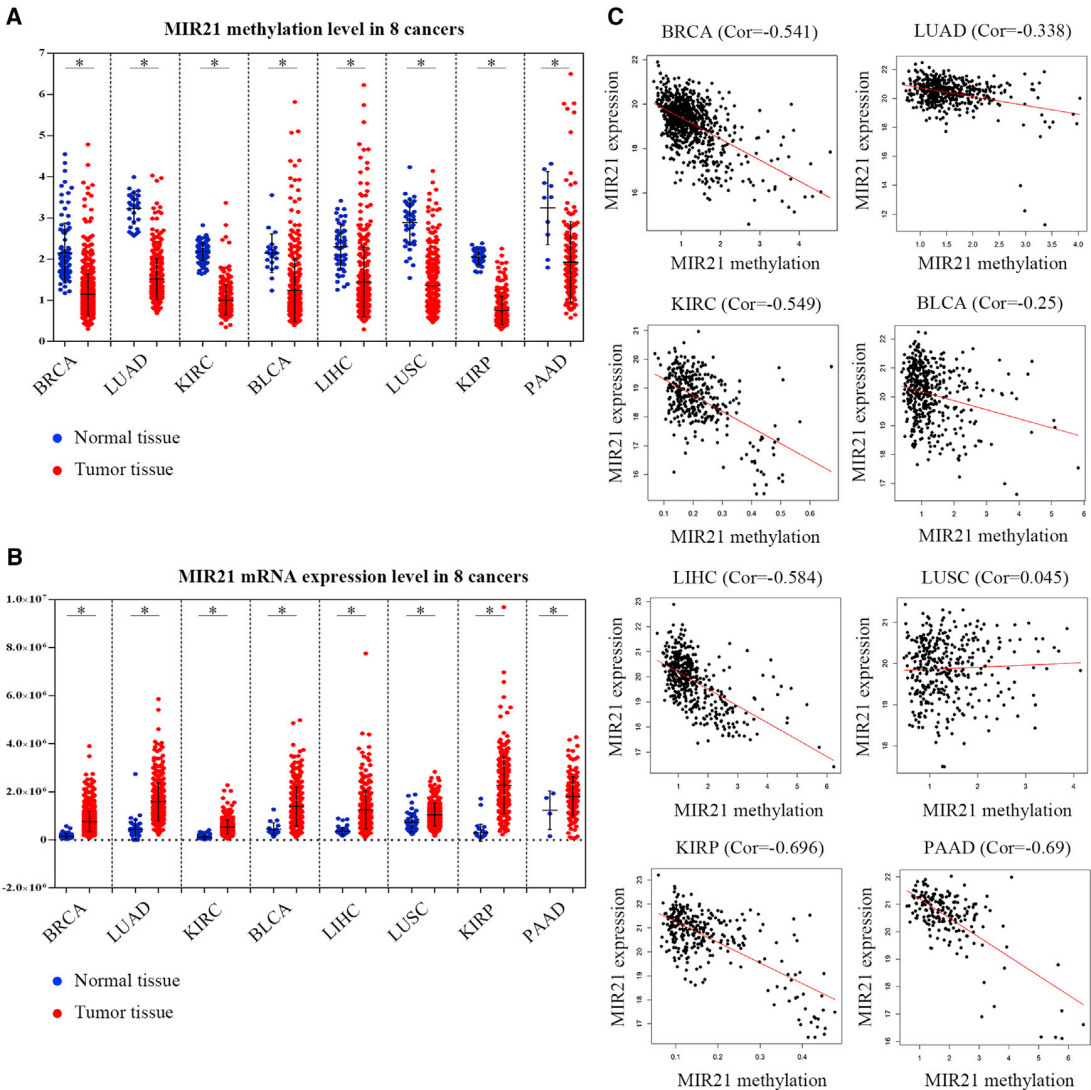
The Correlation between DNA Methylation and miR-21 Expression in Eight Types of Tumors (A) The relative methylation levels of MIR21 in tumor tissues and their normal control tissues. Values shown are means ± standard deviation. (B) MIR21 expression profiles in TCGA (tumor versus normal) samples. (C) Scatterplot of MIR21 expression versus methylation in TCGA-BRCA, TCGA-LUAD, TCGA-KIRC, TCGA-BLCA, TCGA-LIHC, TCGA-LUSC, TCGA-KIRP, and TCGA-PAAD. Cor, coefficient ratio.

The aforementioned data suggest that miR-21 plays an important role in promoting tumor progression. To assess the relationship between miR-21 expression and *MIR21* methylation status, we analyzed eight major cancer types, including breast cancer (BRCA), LUAD, KIRC, bladder urothelial carcinoma (BLCA), LIHC, lung squamous cell carcinoma (LUSC), kidney renal papillary cell carcinoma (KIRP), and PAAD in TCGA data. These cancer types contained sufficient normal and tumor samples for statistical analysis (the number of normal tissue samples is more than 3). The results showed that the total methylation level of the *MIR21* in tumor tissues detected by the 450K methylation chip was significantly lower than the total methylation level of their respective normal control tissues (Figure 2A). Figure 2B shows the expression levels of miR-21 detected by RNA sequencing (RNA-seq) as opposed to the level of methylation. These were significantly upregulated in all tumor tissues compared to their matched normal tissues. In eight types of tumors, we performed a correlation analysis of *MIR21* expression and degree of DNA methylation levels. There is a remarkable negative correlation between *MIR21* expression and degree of methylation in six cancer types (r < −0.3, p < 0.0001; Figure 2C), including BRCA, LUAD, KIRC, LIHC, KIRP, and PAAD. Based on these results, we speculated that high expression of the miR-21 in tumors can be partially attributed to a decrease in the DNA methylation level of *MIR21*.

### CpG Site Methylation in Association with MIR21 Expression

**Figure 3:**
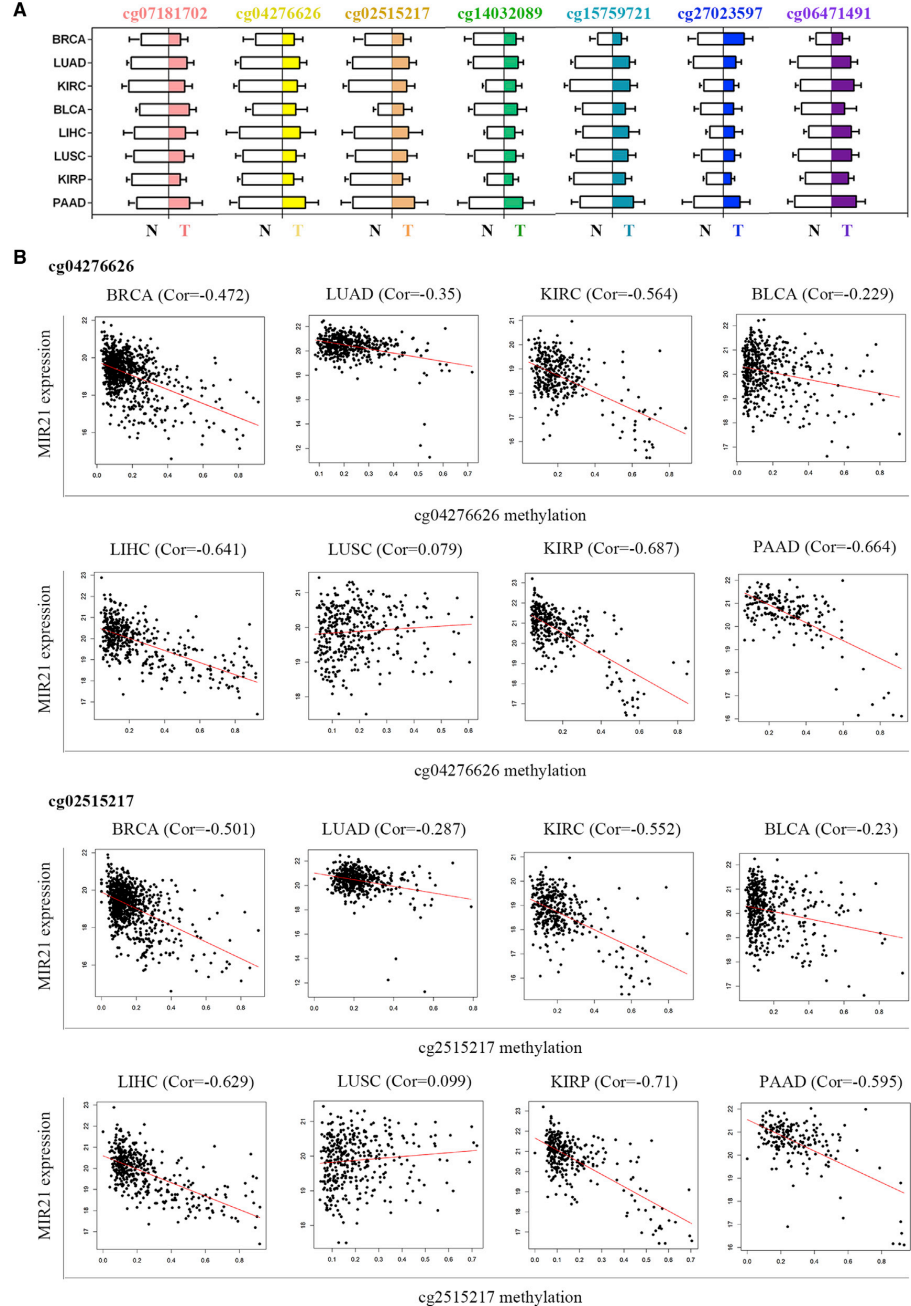
Methylation of CpG Sites and Their Correlation between Methylation and *MIR21* Expression (A) The relative methylation level of seven CpG sites of MIR21 in cancer tissues (T) and their normal control tissues (N). (B) Scatterplot of MIR21 expression versus methylation at probes cg04276626 and cg02515217. Cor, coefficient ratio.

In the 450K methylation detection chip, seven CpG sites were detected in the *MIR21* sequence. Among the eight cancer types, the methylation level of seven sites in tumor tissues was lower than that of the normal control group (Figure 3A). Since the promoter region is the major site of DNA methylation, which regulates gene expression, we focused on cg04276626 and cg02515217 located in TSS200 (the region from the transcription start site [TSS] to 200 nt upstream of the TSS). Figure 3B shows the results of correlation analysis between cg04276626 and cg02515217 methylation and *MIR21* expression in eight cancer types. We found that the methylation levels of cg04276626 and cg02515217 were significantly negatively correlated with *MIR21* expression in BRCA, LUAD, KIRC, LIHC, KIRP, and PAAD (r < −0.3, p < 0.0001).

### CpG Sites in MIR21 Promoter Were Hypomethylated in ccRCC Tissues

**Figure 4:**
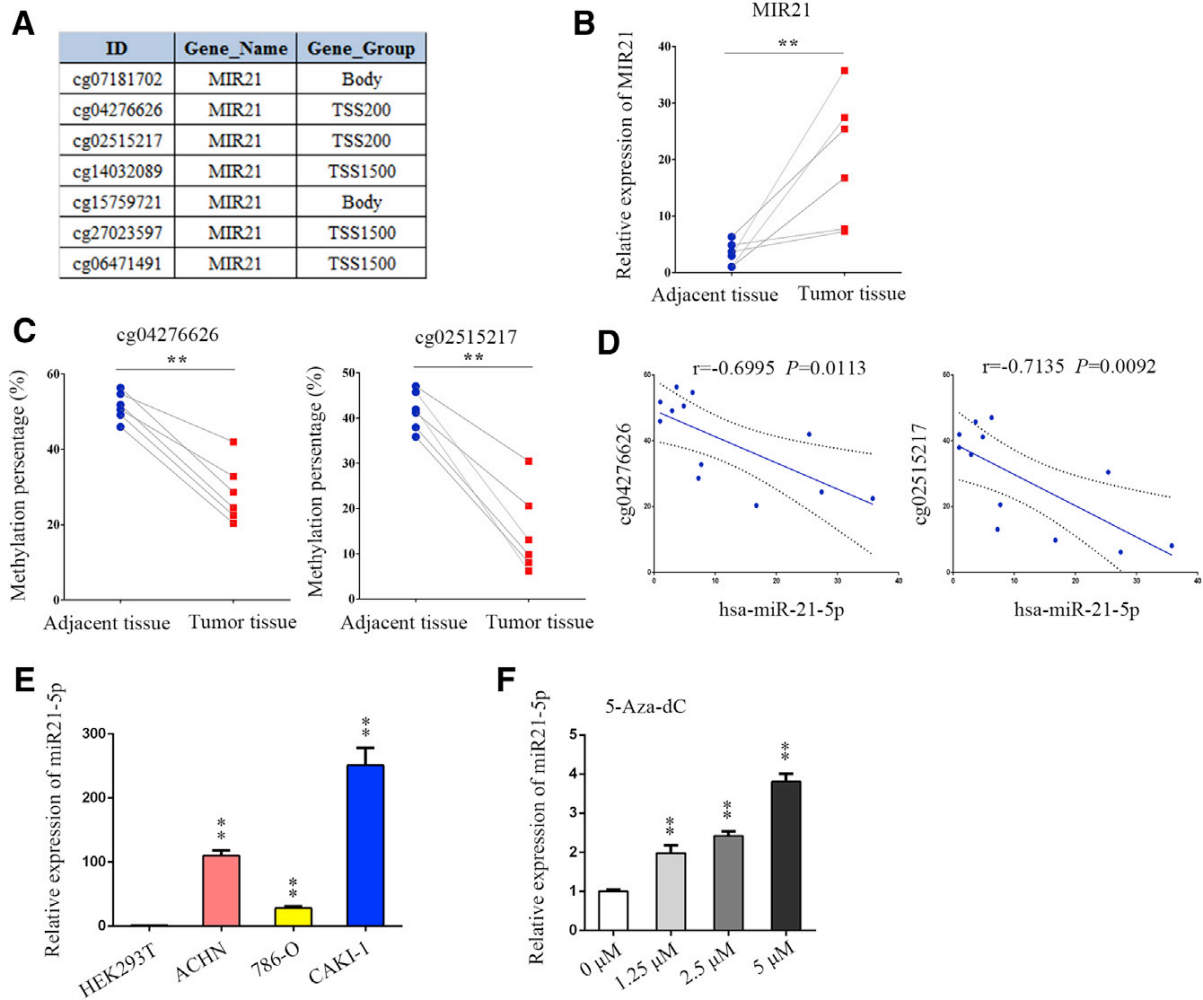
The DNA Methylation Status and *MIR21* Expression Were Further Confirmed by Fresh ccRCC Samples (A) Chart showing all the potential CpG sites of miR-21. (B) hsa-miR-21-5p expression levels in six ccRCC samples compared with the expression in their adjacent non-tumor tissues. ∗∗p < 0.01. (C) Methylation statuses of cg04276626 and cg02515217 were detected by bisulfite genomic sequencing in six paired ccRCC samples. (D) The correlation between hsa-miR-21-5p expression and MIR21 methylation status was analyzed in six paired ccRCC samples. For cg04276626: r = −0.6995, p = 0.0113; for cg02515217: r = −0.7135, p = 0.0092. (E) Relative expression of hsa-miR-21-5p in HEK293T, ACHN, 786-O, and CAKI-1. The experiments were repeated three times. The p value was measured using Student’s t test. ∗∗p < 0.01, compared with that of the HEK293T cells. (F) Relative expression of hsa-miR-21-5p in HEK293T cells, which were treated by different concentrations of 5-Aza-dC (0, 1.25, 2.5, and 5 μM). The p value was calculated for differences between the HEK293T cells treated with 5-Aza-dC and the control cells. The p value was measured using Student’s t test. ∗∗p < 0.01. r, ratio.

Figure 4A is a chart that shows seven CpG sites of the *MIR21* sequence. Our laboratory preserves specimens of ccRCC cancer and adjacent tissues. To validate the results of the bioinformatics analysis, we examined the expression of hsa-miR-21-5p in six pairs of renal clear cell carcinoma samples and the methylation ratio of cg04276626 and cg02515217 sites in each sample. The results showed that hsa-miR-21-5p was highly expressed in tumor tissues in six pairs of tumors and paracancerous samples (Figure 4B).

Using bisulfite sequencing, the cg04276626 and cg02515217 sites of the *MIR21* promoter region in all (6/6) sample tumor tissues were found to be hypomethylated (Figure S1). There were significant differences between tumors and adjacent tissues in the two sites of *MIR21* (Figure 4C). In addition, a significant negative correlation between *MIR21* expression and methylation of two sites was observed in 12 samples (Figure 4D). On the other hand, by detecting hsa-miR-21-5p in the embryonic kidney cells, HEK293T and the three ccRCC cell lines (ACHN, 786-O, and CAKI-1), we found high levels of hsa-miR-21-5p in ccRCC cells (Figure 4E). HEK293T cells with low expression of MIR21 were treated with the demethylating agent 5-Aza-dC to partially restore hsa-miR-21-5p expression (Figure 4F). According to these results, *MIR21* was activated in ccRCC samples partially due to promoter hypomethylation. This study provided evidence for an additional mechanism for regulation of gene expression by epigenetics, where DNA hypomethylation may increase miR-21 expression.

### Expression Patterns of Demethylation-Related Genes

**Figure 5:**
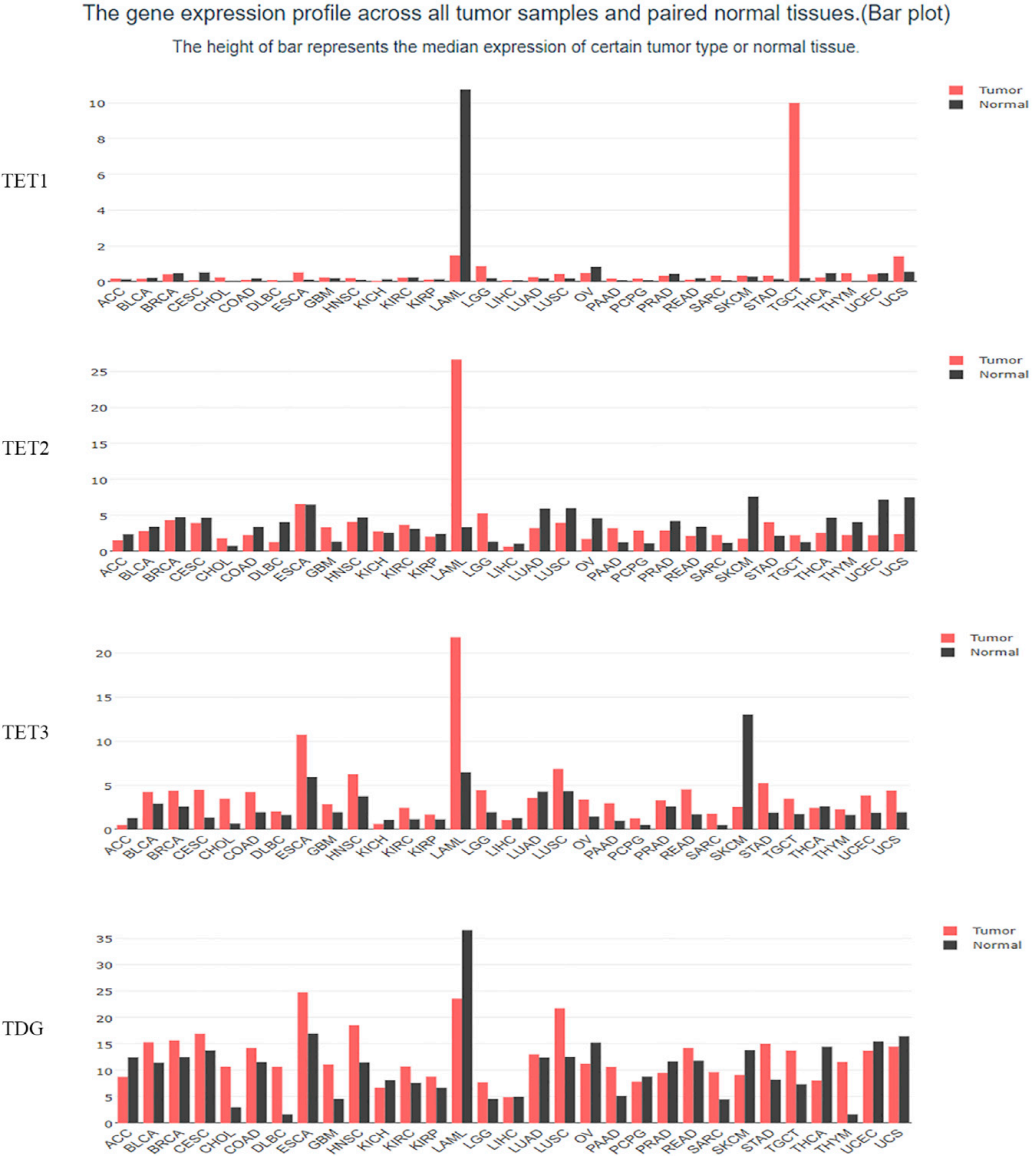
Expression Profiles of TET1, TET2, TET3, and TDG across All Tumors in TCGA

**Figure 6:**
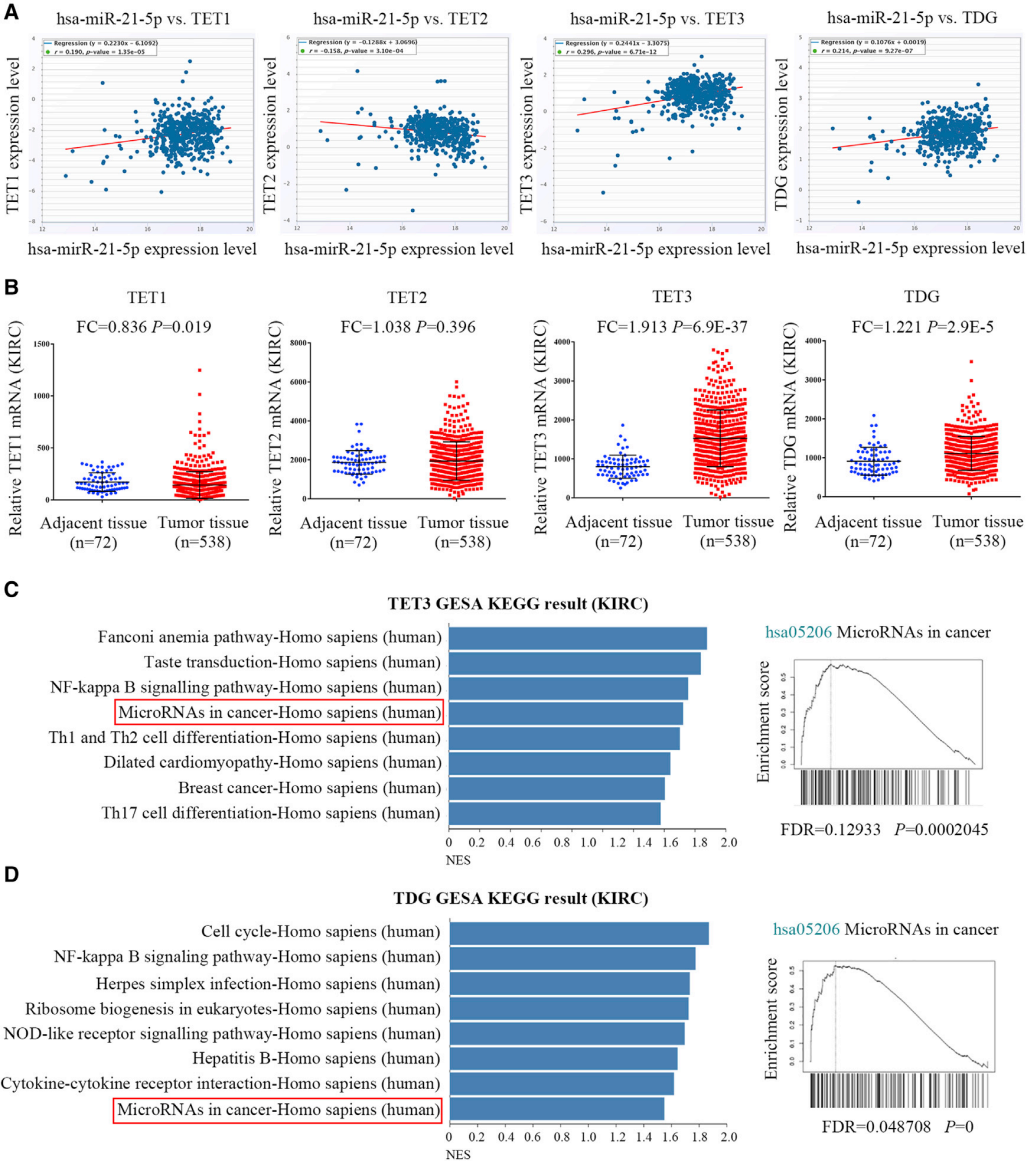
mRNA Expression and GSEA-KEGG Analysis of TETs and TDG (A) Scatterplot of TET1, TET2, TET3, and TDG expression versus hsa-miR-21-5p expression. (B) The relative expression levels of TET1, TET2, TET3, and TDG in TCGA-KIRC tissues and normal tissues. (C) LinkedOmics GSEA KEGG analysis of TET3 co-expression genes in TCGA-KIRC samples. (D) LinkedOmics GSEA KEGG analysis of TDG co-expression genes in TCGA-KIRC samples.

DNA demethylation is the conversion of methylcytosine to hydroxymethylcytosine under the action of the T5-methylcytosine hydroxylase (TET) family. Finally, the combination of thymine DNA glycosylase (TDG) and base excision repair (BER) can remove 5-formylcytosine (5fC) and 5-carboxycytosine (5caC) from DNA, producing unmodified cytosine.^12^ To investigate whether demethylation affects *MIR21* expression, we analyzed the expression of TET1, TET2, TET3, and TDG in tumors and the correlation between TET-TDG and *MIR21* expression in TCGA. According to multi-tumor data analysis, TET3 and TDG are highly expressed in most tumor types (Figure 5). For example, in renal clear cell carcinoma, the expressions of TET1, TET3, and TDG are positively correlated with that of hsa-miR-21-5p (Figure 6A). TET3 and TDG were significantly highly expressed in tumor tissues (Figure 6B). For renal clear cell carcinoma samples, gene set enrichment analysis (GSEA) Kyoto Encyclopedia of Genes and Genomes (KEGG) pathway enrichment showed a positive correlation between expressions of TET3/TDG and miRNAs in cancer (Figures 6C and 6D). These results suggested that elevated expression of the demethylases TET3 and TDG is a possible cause of elevated miR-21 expression.

### TET3 and TDG Knockdown Increased miR-21 Expression

**Figure 7:**
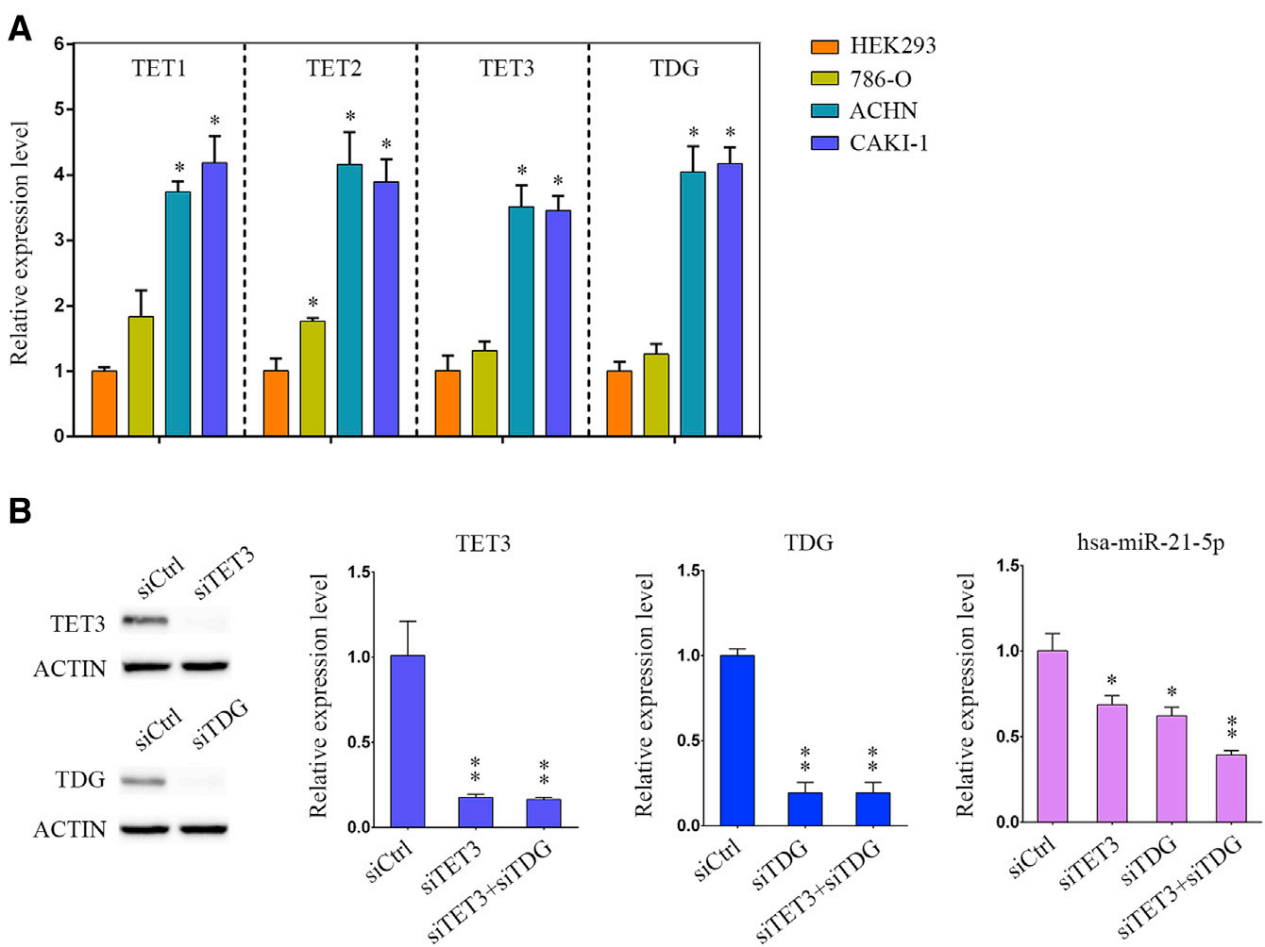
Knockdown of TET3 and TDG Downregulated the Expression of hsa-miR-21-5p (A) The expressions of TET1, TET2, TET3, and TDG were examined by quantitative PCR in HEK293, 786-O, ACHN, and CAKI-1 cells. The p value was measured using Student’s t test. ∗p < 0.05, compared with that of the HEK293 cells. (B) TET3 and TDG siRNA sequences were used to knock down TET3, TDG, or both of them in ACHN cells. The expressions of TET3, TDG, and hsa-miR-21-5p were detected by quantitative PCR. The p values were calculated for differences between the ACHN cells transfected with TET3 or TDG-specific siRNA sequences and the control siRNA sequences (siCtrl) and measured with Student’s t test. ∗p < 0.05; ∗∗p < 0.01.

We quantitatively detected expressions of four demethylase genes in three ccRCC cell lines (786-O, ACHN, and CAKI-1). TET1, TET2, TET3, and TDG were all highly expressed in ACHN cells (Figure 7A). To investigate whether TET3 and TDG affect *MIR21* expression, we used TET3 and TDG small interfering RNA (siRNA) sequences to knock down TET3, TDG, or both in ACHN cells. Quantitative PCR detection revealed that the combination of TET3 and TDG siRNAs markedly decreased the expression of hsa-miR-21-5p, whereas single modification of TET3 or TDG moderately affected the expression of hsa-miR-21-5p in ACHN cells (Figure 7B). Similar results were observed in CAKI-1 cells (data not shown). These data indicated that intracellular *MIR21* expression is regulated by TET3 and TDG.

### cg02515217 Locus Is in a Conserved Binding Sequence of Transcription Factors

**Figure 8:**
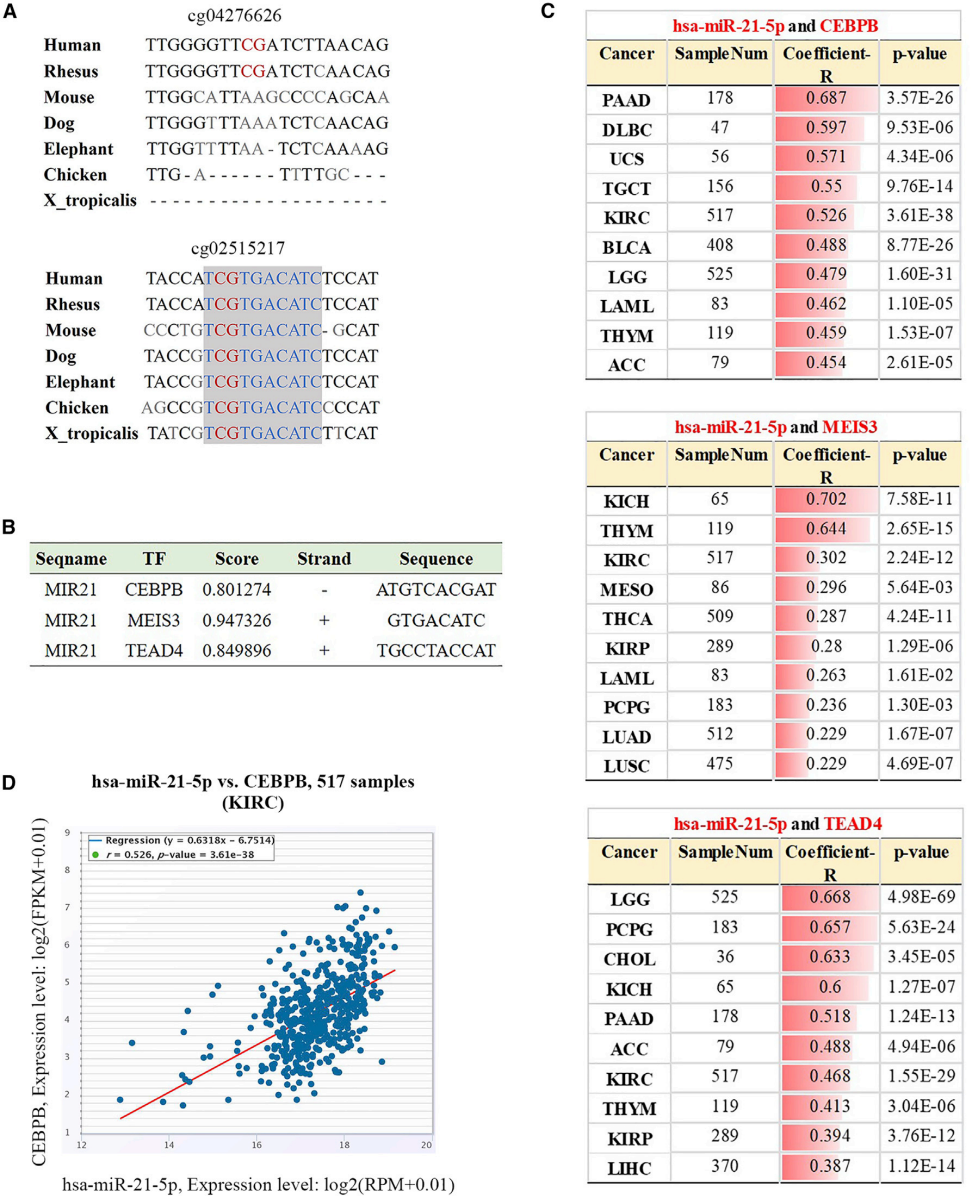
cg02515217 Locus Is Located in a Binding Sequence of Transcription Factors (A) Positions −135 to −116 (cg04276626) and −33 to −14 (cg02515217) in *MIR21* gene promoter. (B) Lists of three transcription factors for which the binding sequences are located near the cg02515217 locus. (C) starBase analysis of the correlation between hsa-miR-21-5p and CEBPB, MEIS3, and TEAD4 expressions in TCGA tumors. (D) starBase also revealed a significant positive correlation between hsa-miR-21-5p and CEBPB in TCGA-KIRC samples. r = 0.526, p = 3.61e−38.

Demethylation of the promoter region of genes generally promotes transcription factor binding, thereby initiating gene transcription.^13^ We analyzed the conservation of the cg04276626 and cg02515217 loci and found that the sequence near cg02515217 is highly conserved in a variety of animals (Figure 8A). Then, we found that transcription factors CEBPB, MEIS3, and TEAD4 bind to, or in the vicinity of, cg02515217 loci (Figure 8B). CEBPB, MEIS3, and TEAD4 were co-expressed with has-miR-5p in cancers. Among them, the co-expression analysis of CEBPB and miR-21 exhibited the highest coefficient ratio value (Figure 8C, Coefficient-R). For example, in ccRCC, the ratio (r in Figure 8D) of the co-expression analysis of CEBPB and hsa-miR-21-5p was 0.526 (p < 0.0001).

## Discussion

The discovery that miR-21 upregulation is associated with gene hypomethylation could further demonstrate the major role of epigenetics in the regulation of cancer gene expression. In this study, we found that miR-21 is highly expressed in 15 cancer types and that high levels of hsa-miR-21-5p decreased the overall survival in six kinds of cancer patients. The *MIR21* methylation level in eight types of tumors analyzed in this study was lower in tumor tissues than that in the normal control group. Correlation analysis showed that *MIR21* hypomethylation is associated with the increase in gene expression. Two of the CpG loci located within the 200-bp positions before the transcription initiation site were found to play an important role in the expression of *MIR21*. We confirmed *MIR21* hypomethylation in tumor tissues by methylation sequencing in fresh renal clear cell carcinoma samples, whereas the hsa-miR-21-5p level was increased as detected by miRNA quantitative PCR. The use of a demethylating agent in HEK293T cells increased hsa-miR-21-5p level, indicating that the increased expression of demethylase genes *in vivo* may promote *MIR21* expression. TCGA data analysis revealed that TET3 and TDG increased expression in most types of tumors. The expression of miR-21 was decreased in TET3/TDG knockdown cells. As demethylation of the CpG sites in the promoter region may facilitate transcription factor binding, our analysis also showed that the sequence near the cg02515217 locus is a conserved sequence of transcription factor binding. The transcription factors CEBPB, MEIS3, and TEAD4, which bind to the sequence of the cg02515217 locus region, were co-expressed with miR-21 in tumors. The results of this study increase the knowledge required for investigating the molecular regulation of miR-21.

Previous studies have reported the relationship between miRNA promoter region methylation and mature miRNA expression levels.^14^, ^15^, ^16^ miR-9 expression is downregulated in human colorectal cancer cell lines. Methylation-specific polymerase chain reaction (MSP) and sulfite-sequencing analysis revealed that miR-9 (especially miR-9-1) expression is inversely proportional to the methylation level of the CpG island of the gene promoter region.^16^ In malignant breast cancer cells, miR-203 was silenced by hypermethylation of the promoter region of the gene and promoted tumor cell growth and invasion.^17^ miR-142 upregulation is significantly correlated with hypomethylation of the gene promoter in the autistic brain.^18^ Our study explained the relationship between *MIR21* demethylation and gene expression in multiple tumors based on TCGA data analysis, indicating that *MIR21* hypomethylation level is an important cause of increase in mature miR-21 level in tumors.

In mammals, 5-methylcytosine (5mC) is the main form of DNA modification. 5mC is chemically and genetically stable.^19^^,^^20^ Despite its stability, the 5mC form of DNA methylation can be converted to unmodified cytosine, and mediated by TET dioxygenase to oxidize 5mC to 5-hydroxymethylcytosine (5hmC), 5-A acylcytosine (5fC), and 5-carboxycytosine (5caC), followed by thymidine DNA glycosylase (TDG)-dependent BER.^12^^,^^21^ Our results showed an increase in the expression of the demethylases TET3 and TDG in tumors and demonstrated that TET3 and TDG knockdown reduced miR-21 expression in ccRCC cells. We will further investigate the expression of TET3 and TDG in cancer and their effects on gene expression profiles in subsequent studies.

Our results also indicated that the sequence near the CpG site in the *MIR21* promoter region is the binding sequence of the transcription factors, including CEBPB, MEIS3, and TEAD4. McClure et al.^22^ have reported that CEBPB and STAT3 synergize to bind and activate the promoters of miR-21 in naive Gr1+CD11b+ cells. We provided an alternative binding sequence for CEBPB and STAT3 on *MIR21* promoter. MEIS3 and TEAD4 play critical roles during embryonic development.^23^^,^^24^ TEAD4 is an evolutionarily conserved transcription factor in the Hippo signaling pathway. TEAD4 binds to almost the same regions of YAP1 in the mammalian genome.^25^ Recently, the role of TEAD4 protein in tumorigenesis has been reported.^26^^,^^27^ TEAD4 regulates epithelial-to-mesenchymal transition and metastasis in a YAP-independent manner in colorectal cancer.^28^ Our study provided a possible cancer-promoting mechanism for TEAD4. The regulation of the expression of mir-21 by MEIS3 and TEAD4 also requires subsequent experiments to confirm.

Taken together, our paper suggests that gene hypomethylation plays an important role in upregulating *MIR21* expression in tumors. The demethylases TET3 and TDG are involved in the hypomethylation of *MIR21* DNA. Hypomethylation of the promoter region may facilitate transcription factor binding and lead to overexpression of miR-21.

## Materials and Methods

### miR-21 Expression and Survival Results

miR-21 differential expression analysis results integrated by TCGA were downloaded from starBase v3.0 (2018) (http://starbase.sysu.edu.cn/index.php). The Kaplan-Meier plots of Figure 1C were also downloaded from starBase.

### Analysis of TCGA Data

miRNA sequencing (miRNA-seq), DNA methylation (Illumina Human Methylation 450), and clinical data were downloaded from TCGA (https://portal.gdc.cancer.gov/)^29^ from April 2018 to October 2018. The microRNA-Seq data files were normalized and transferred into a txt file using a Perl script. Relative gene expression of *MIR21* was reflected by the intensity of the probe signal and plotted using GraphPad Prism 6. Single-gene methylation information was extracted from the fasta file using a custom Perl script. We screened samples for both expression and methylation data. The correlation between DNA methylation/loci methylation level and *MIR21* expression was analyzed by Spearman’s test.

### Quantitative PCR

Real-time quantitative PCR was used to monitor cells and six pairs of samples (tumor samples and their adjacent non-tumor tissues) from patients with ccRCC. For mRNA detection, total RNA from tissues was extracted using a reagent (TRIzol, Invitrogen, Thermo Fisher Scientific), and cDNA was synthesized using the RevertAid First Strand cDNA Synthesis Kit (Thermo Fisher Scientific). Primer sequences for human *TET1*, *TET2*, *TET3*, *TDG*, and *ACTB* are shown in Table S1. *ACTB* was used as an internal control in these experiments. For miRNA detection, cDNA was synthesized from total RNA using the Taqman MicroRNA Reverse Transcription Kit with specific primers of hsa-miR-21-5p (Applied Biosystems). *RNU6-6P* was used as an internal control in these experiments. All PCR reactions were performed on ABI PRISM 7900 PCR System (Applied Biosystems) using Taqman Universal PCR Master Mix (Applied Biosystems), following the manufacturer’s protocol. The amplification protocol included an initial denaturation step at 95°C for 10 min, followed by 45 cycles at 95°C for 15 s and 60°C for 60 s. Relative quantification of miRNA expression was calculated using the 2^−ΔΔCt^ method.^30^

### Methylation Pyrosequencing

Pyrosequencing was used to verify differences in degree of DNA methylation at the two CpG sites of the miR-21 promoter in six paired ccRCC patient samples (six males), aged between 48 and 75 years. The use of human tissues was approved by the Fuzhou General Hospital Institutional Review Board (IRB) (Fuzhou, China) with written consent (approval no. 2013-017). All the tissue detections were conducted following the receipt of informed written consent from the patients. DNA samples were submitted to Shanghai Geneland Biotech and processed with the following procedure. Each individual’s DNA was treated with sodium bisulphite using an EpiTect Bisulfite Kit (QIAGEN), according to the manufacturer’s recommendations. Then, the treated DNA was amplified by a bisulphite-polymerase chain reaction. Quantitative DNA methylation of each CpG was analyzed with the PyroMark Q96 ID pyrosequencing instrument (QIAGEN).^31^

### Analysis of Demethylase and Its Correlation with hsa-miR-21-5p

The results of expression analysis of demethylase in multiple tumors were generated from the GEPIA (http://gepia.cancer-pku.cn).^32^ Then, we downloaded the correlation data between hsa-miR-21-5p expression and demethylase expression (TET1, TET2, TET3, and TDG) integrated by TCGA from starBase v3.0 (2018) (http://starbase.sysu.edu.cn/index.php).^33^

### Prediction of Transcription Factors Bound at Methylation Sites

The 1,000-bp sequence of the *MIR21* promoter region was downloaded from the UCSC genome browser (https://genome.ucsc.edu/cgi-bin/hgTracks).^34^ The transcription factors that were predicted to be bound to the promoter region of *MIR21* were analyzed using JASPAR (http://jaspar.genereg.net).^35^

### Cell Culture

HEK293T cells were purchased from the Shanghai Cell Bank of the Chinese Academy of Sciences (Shanghai, China). HEK293T cells were cultured in DMEM high-glucose medium (Thermo Fisher Scientific, Waltham, MA, USA) containing 10% (v/v) fetal bovine serum (FBS) (Thermo Fisher Scientific, Waltham, MA, USA) and 1% penicillin/streptomycin (Thermo Fisher Scientific, Waltham, MA, USA). Three ccRCC cell lines, 786-O, CAKI-1, and ACHN (GeneChem, Shanghai, China) were grown in RPMI-1640 medium, supplemented with 10% FBS and 1% penicillin/streptomycin. All the cells were cultured at 37°C in a humidified incubator under 5% CO_2_.

### Knockdown of TET3 and TDG mRNA in Renal Cancer Cells

Clear cell renal carcinoma ACHN cells were grown in 6-well plates and transfected with TET3 siRNA (HIPPOBIO, Xiamen, China), TDG siRNA (HIPPOBIO, Xiamen, China), or negative control siRNA (si-control, siCtrl; HIPPOBIO, Xiamen, China) at a concentration of 100 pmol per well. Cell transfection was performed with Lipofectamine RNAiMAX Transfection Reagent (Thermo Fisher Scientific, Waltham, MA, USA). The knockdown effect of siTET3 and siTDG was examined by real-time RT-PCR using RNA extracted 24 h after transfection. Then, *MIR21* expression was analyzed by quantitative PCR within siCtrl-, siTET3- or siTDG-transfected ccRCC cells as previously described. The siRNA sequences are shown in Table S2.

### Statistical Analysis

Experimental data were collected from at least three technical replicates and expressed as the mean ± standard deviation. Statistical differences between the groups were analyzed with the unpaired Student’s t test or one-way ANOVA with the Bonferroni correction for multiple comparisons. The data were graphically displayed using GraphPad Prism 6 software and R. p value <0.05 indicated a statistically significant difference. Kaplan-Meier analysis of overall survival for patients was conducted based on all available data and compared with the log-rank test.

## Author Contributions

J.L. and R.X. designed the experiments. T.T., L.Z., and H.D. performed the experiments. J.L. provided the patient samples. J.L. and T.T. analyzed the data. J.L. wrote the manuscript.

## Conflicts of Interest

The authors declare that they have no competing interests.

## Supplemental Information

Document S1. Tables S1 and S2 and Figure S1

Document S2. Article plus Supplemental Information
