# Supplementary material for: Hypomethylation Causes MIR21 Overexpression in Tumors
Source: Mol Ther Oncolytics. 2020 May 26;18:47–57. doi: 10.1016/j.omto.2020.05.011 (PMC7321816; doi:10.1016/j.omto.2020.05.011)
Supplement: Document S1. Tables S1 and S2 and Figure S1 [file mmc1.pdf]

OMTO, Volume *18*

Supplemental Information

Hypomethylation Causes

MIR21 Overexpression in Tumors

Jun Lu, Ting Tan, Ling Zhu, Huiyue Dong, and Ronghua Xian

Table S1. Details of primers used for gene expression analysis and their expected product size

Target gene	Forward Primer (5' to 3')	Reverse Primer (5' to 3')	Amplicon (bp)
h*TET1*	AGTAAGCCTTCGTCACTGCC	TGAGGCTGTTGCGTCATTCT	23
h*TET2*	GAGCAGGTCCTAATGTGGCA	CTTCTGCGAACCACCCACTT	154
h*TET3*	ATCAGAACCAGGTGACCAACG	GCATTCTGGTTCTCCTCGCT	277
h*TDG*	CAGCCCCATAAGATTCCAGA	ATCCTCTTGGGCAAGCTGTA	207
h*Actin*	AGCGAGCATCCCCCAAAGTT	GGGCACGAAGGCTCATCATT	285

Table S2. siRNA sequences

Target gene	Sense sequence (5' to 3')	Antisense sequence (5' to 3')
siTET3	GCAGUUUGAGGCUGAAUUUGG	AAAUUCAGCCUCAAACUGCCG
siTDG	GCCCAAGACAAAGUUCAUUTT	AAUGAACUUUGUCUUGGGCTC
siCtrl	GUGAGCGUCUAUAUACCAUDT	AUGGUAUAUAGACGCUCACDT

Figure S1-1

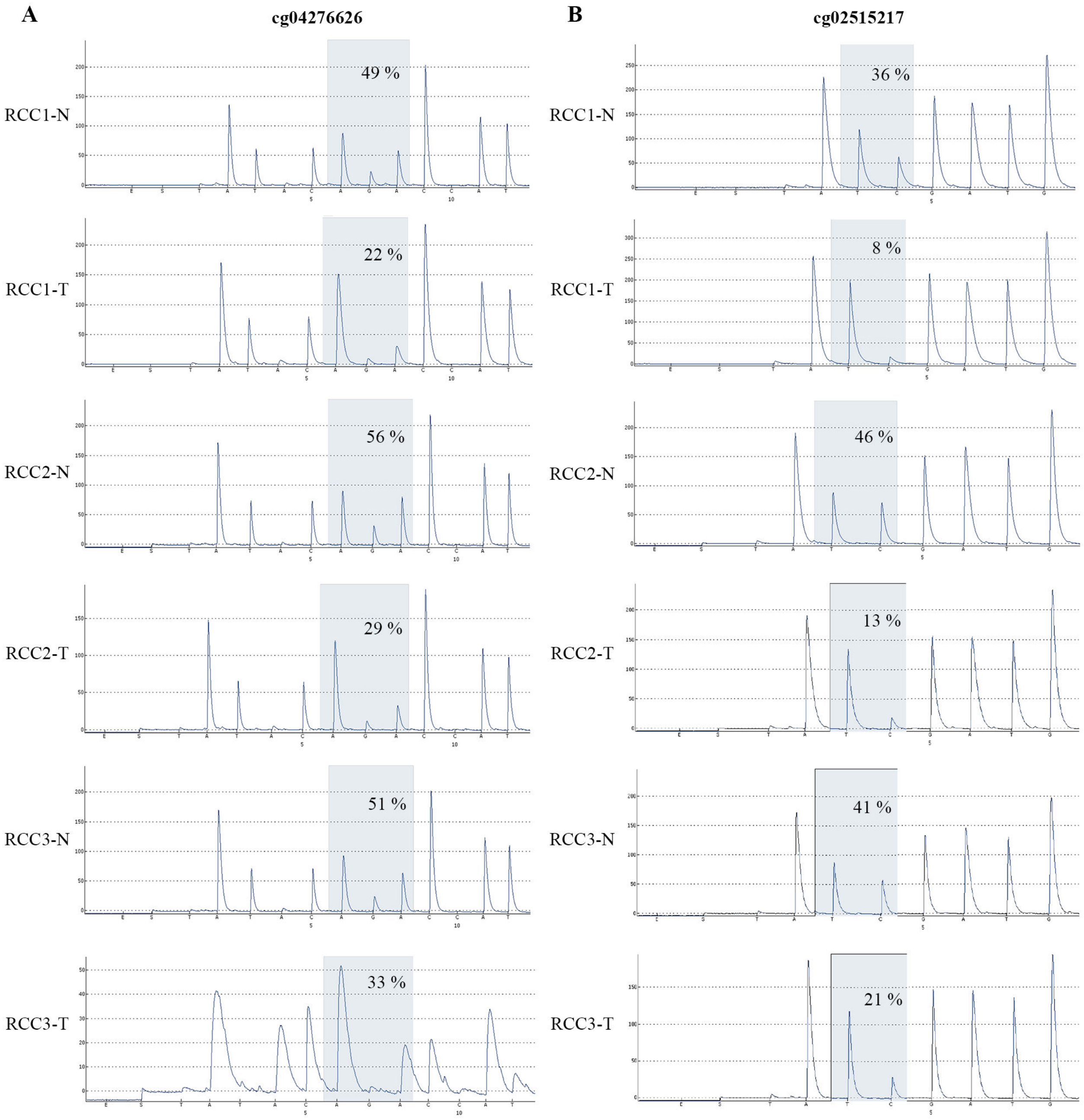
A
cg04276626
RCC1-N
49 %
RCC1-T
22 %
RCC2-N
56 %
RCC2-T
29 %
RCC3-N
51 %
RCC3-T
33 %
B
cg02515217
RCC1-N
36 %
RCC1-T
8 %
RCC2-N
46 %
RCC2-T
13 %
RCC3-N
41 %
RCC3-T
21 %

Figure S1-2

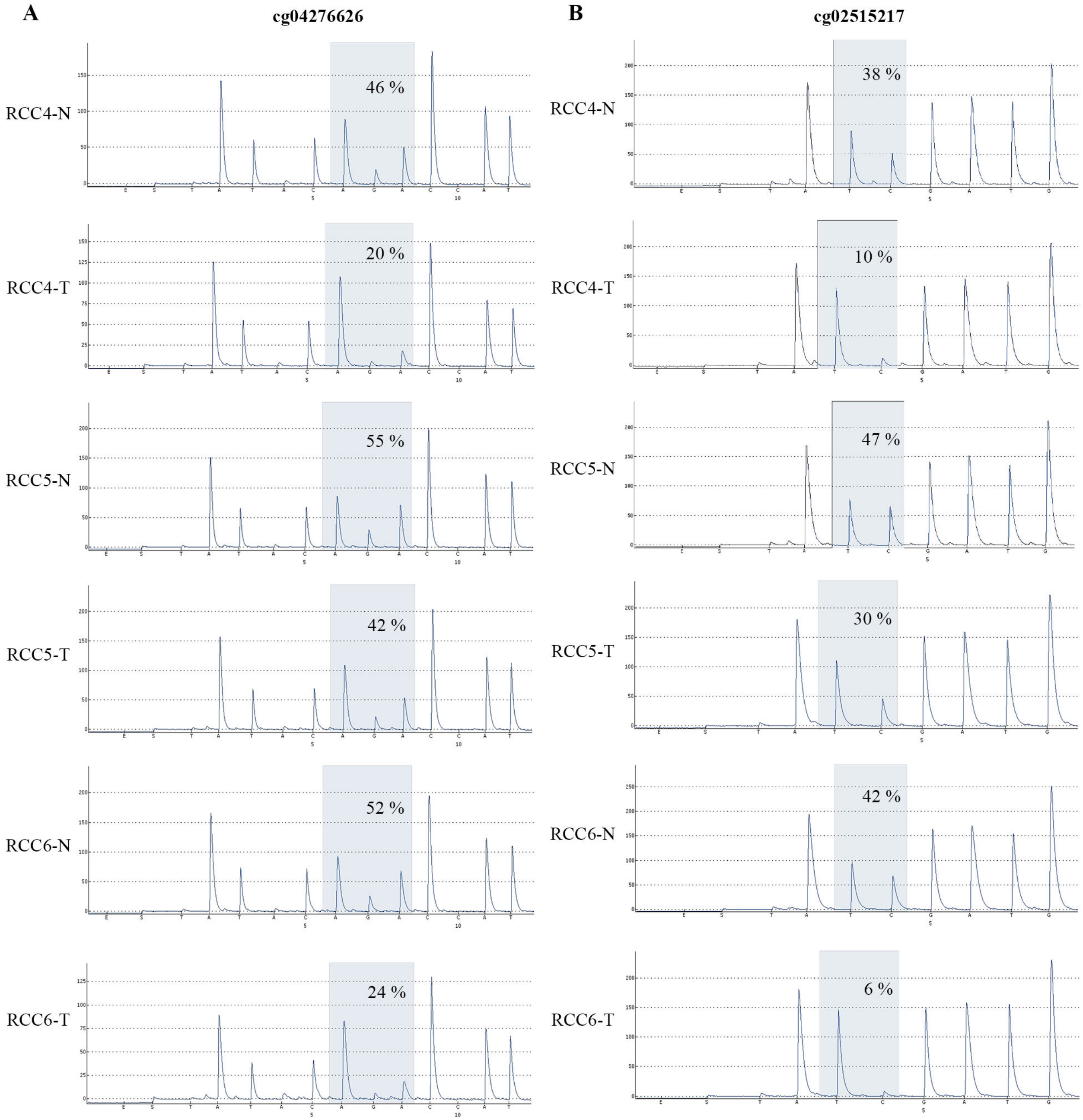

A
cg04276626
RCC4-N
46 %
RCC4-T
20 %
RCC5-N
55 %
RCC5-T
42 %
RCC6-N
52 %
RCC6-T
24 %
B
cg02515217
RCC4-N
38 %
RCC4-T
10 %
RCC5-N
47 %
RCC5-T
30 %
RCC6-N
42 %
RCC6-T
6 %

Supplementary Figure Legend:

Figure S1. *MIR21* DNA methylation levels in human ccRCC samples. (A-B) The cg04276626 and cg02515217 sites of the *MIR21* promoter region in six paired of tumor and adjacent tissue samples were detected using the bisulfite sequencing.
